# Supplementary material for: A study of the impact of urban business credit environment on environmental pollution
Source: PLoS One. 2025 Jan 7;20(1):e0310636. doi: 10.1371/journal.pone.0310636 (PMC11706382; doi:10.1371/journal.pone.0310636)
Supplement: Attachment — Submitted filename: PLOSONE_review.docx [file pone.0310636.s002.docx]

Dear author(s),

I had the opportunity to read and revised your article.

The manuscript investigates the relationship between urban business credit environment and environmental pollution in 276 Chinese cities from 2010 to 2021. The study utilized the entropy method to calculate the environmental pollution index and employs the CEI index to describe the urban business credit environment. Moreover, it employs a spatial Durbin model to analyze the mechanism and transmission path of the urban business credit environment on environmental pollution.

I believe your work coherent as the Journal “PLOS ONE”, regarding an interesting topic not fully investigated in the literature until now. However, I have some suggestions and minor concerns that may be improve the quality of your work.

- Starting from a general suggestion, I recommend you to carefully read your work, since there are several typos, punctuation mistakes with too long period sentences. I list some examples that I found in the text. Lines 52-59. Line 79. Line 91-96. Line 98. Line 144. Line 214. Etc.
- I suggest adjusting the Introduction section, since it is too long and somewhere a bit confused. I recommend to better highlight your contribution, with the research questions, the aim and the structure of the manuscript in a logic order. Moreover, you should include some missing references: lines 52-53; lines 100-104.
- The study relies on data from 276 cities over a specific period (2010-2021). If it is possible (considering availability of data) extending the analysis period beyond 2021 and incorporating more recent data could help validate the findings and account for any changes in trends over time.
- Although the spatial Durbin model is adequate for your research because it is useful for capturing spatial relationships, it may not fully account for all relevant variables influencing environmental pollution, such as local policy changes, industrial shifts, or unobserved regional characteristics.
- The study could improve by addressing potential endogeneity issues. You might consider including instrumental variables or difference-in-differences approach that could help establish causality more convincingly.
- I suggest to better describe the collection of data and the database used. For example, regarding the technological innovation, which kind of database did you used and which kind of patent?
- Moreover, the study focuses only on Chinese cities, which may limit the generalizability of the findings to other contexts. Different countries or regions might exhibit different relationships between business credit environments and environmental pollution due to varying regulatory, economic, and social conditions. Therefore, I suggest considering this aspect mentioning it at the end of the manuscript in the Conclusion section as future directions and further investigations.
- The article suggests that improving the urban business credit environment can eventually lead to reduced environmental pollution. However, the policy recommendations could be more detailed, outlining specific measures that cities can take to balance business credit improvements with environmental sustainability.
- Finally, I suggest mentioning the limitation/limits of the study.

With best wishes in the publishing process.
