# Supplementary material for: A study of the impact of urban business credit environment on environmental pollution
Source: PLoS One. 2025 Jan 7;20(1):e0310636. doi: 10.1371/journal.pone.0310636 (PMC11706382; doi:10.1371/journal.pone.0310636)
Supplement: Attachment — Submitted filename: Review of manuscript_PONE-D-24-15225.docx [file pone.0310636.s003.docx]

Review of manuscript "A study of the impact of urban business credit environment on environmental pollution" (PONE-D-24-15225). The study highlights a positive spatial association between urban pollution and commercial credit indices in China; with an inverted "U" shape relationship indicating that improving the urban business credit environment can reduce pollution once a threshold is reached. Regional variations exist in the impact of business credit on environmental deterioration, emphasizing the need for tailored strategies to strengthen commercial credit systems and promote environmental sustainability in different regions.

- More detailed suggestions and comments are presented as follows:

1. Line 53 – 55: Previous research has demonstrated the significant influence social credit plays in improving the urban environment. Please cite the related research articles for in-depth understanding.
2. Section 1 Introduction: The section is quite lengthy and splitting the section into various sub-sections such as Introduction, Literature Review, Research gap, Scope of the study, and Research questions will be more helpful.
3. In the introduction, you need to connect the current state of research to your paper goals. Please follow the literature review by a clear and concise state of the art analysis. This should clearly show the knowledge gaps identified and link them to your paper goals. Also, please provide the organization of the paper in the last paragraph of the introduction.
4. Section 4 Empirical results and analysis: Would you consider splitting the fourth section, into two parts, Results and Discussion? At present, the length of the results section is too long, which is not conducive to grasping the key points.
5. Section 5 Conclusion and policy implications are too long, and it is recommended that the main conclusions be distilled and streamlined.
6. The sub-section 5.2 Policy Recommendations: It can be included in the separate sub-section into Discussion section.
