# Supplementary material for: A study of the impact of urban business credit environment on environmental pollution
Source: PLoS One. 2025 Jan 7;20(1):e0310636. doi: 10.1371/journal.pone.0310636 (PMC11706382; doi:10.1371/journal.pone.0310636)
Supplement: Attachment — Submitted filename: Response to Reviewers.docx [file pone.0310636.s004.docx]

**Revision Report**

**Dear Editors and Reviewers:**

Thank you for your letter and for the reviewers' comments concerning our manuscript entitled “A study of the impact of urban business credit environment on environmental pollution” (ID: PONE-D-24-15225). Those comments are all valuable and very helpful for revising and improving our paper, as well as the important guiding significance to our research.

We have studied comments carefully and have made corrections which we hope meet with approval. We tried our best to improve the manuscript and made some changes in the manuscript. These changes will not influence the content and framework of the paper. The summary of corrections and the responses to the reviewer’s comments are as follows. The original comments by reviewers are in blue, and the responses are shown in black. Revised portions are marked in red on the paper.

Your efforts in reviewing the manuscript are highly appreciated.

Once again, we would like to express our sincere gratitude for your thorough review and invaluable feedback. Your detailed comments and suggestions have significantly contributed to enhancing the quality of our manuscript. We greatly appreciate the time and effort you have invested in providing us with constructive guidance. Your insights have been instrumental in refining our work, and we are grateful for your support.

Best Regards.

Yours sincerely,

Jing Wu, Qing Guo

30, July, 2024

**Summary of the revision:**

**Section 1 (Introduction):** In the revision of this section, we restructured the lines of the introduction in accordance with the reviewers' comments, dividing the section into subsections such as Introduction, Literature Review, Research Gaps, Scope of the Study, and Organizational Structure. The missing literature was fully added to the original first draft, and the organizational structure of the paper was added to the final section.

**Section2 (Theoretical analysis and research hypothesis):** In order to avoid the structural confusion caused by the excessive length of Section 4 of the original draft, the modification of this section is mainly to place the theoretical elaboration of the mediated effects model in Section 4 in this section as the third theoretical hypothesis point of this paper.

**Section 3 (Methodology and description of variables):** There are two main changes in this chapter:(1) A more detailed description of the research methodology of this paper. At the suggestion of reviewer 4, we introduce relevant diagrams and theories to develop a detailed explanation of the spatial Durbin model construction process in the original manuscript. (2) The sources and citations of the data in this paper have been scientifically explained. At the suggestion of two reviewers, we have described the data sources and citations by referring to specific URLs.

**Section4 (Empirical analysis and discussion):** The main changes in this chapter are the following:(1) In order to see more intuitively the relationship between urban business credit environment and environmental pollution, this section combines the original benchmark regression and spatial Durbin model empirical results together for overall interpretation, which also well avoids the length of the chapter. (2) This section adds an explanation of the spatial and temporal changes in the urban business credit environment and environmental pollution. In order to react to the spatial regional characteristics of the dependent and independent variables. (3) An endogeneity explanation is added to justify this study. (4) In order to avoid the confusing structure of the original manuscript, this revision divides this chapter of the original manuscript into two parts: results and discussion, which makes the line clearer.

**Section5 (Conclusions and research recommendations):** The main changes in this chapter are the following:

(1) This revision focuses on the streamlining of the main conclusions as well as a more refined approach to initiatives related to policy recommendations. (2) Limitations of the paper have been added.

In addition, the manuscript has been carefully revised considering the language and grammar problems, including the sentences that are convoluted and hard to follow.

Respond to the editor's comments

1. Comment:

Dear Dr. Wu,

Thank you for submitting your manuscript to PLOS ONE. After careful consideration, we feel that it has merit but does not fully meet PLOS ONE’s publication criteria as it currently stands. Therefore, we invite you to submit a revised version of the manuscript that addresses the points raised during the review process.

1. Reply: Thank you for your thorough review and valuable suggestions for my manuscript. Your meticulous examination and professional insights have greatly contributed to the improvement and enhancement of this paper in multiple aspects. Your recommendations have not only helped us delve deeper into the theoretical and methodological aspects but also made the structure and presentation of the article more rigorous and clearer.

I deeply appreciate the time and effort you have dedicated to this review, and I am pleased to have benefited from your feedback. Your suggestions have been crucial in elevating the quality and academic contribution of the paper. We hope that these improvements will enable the manuscript to better meet the journal's standards and provide valuable insights for research in the related field.

Once again, thank you for your careful review and invaluable feedback. I hope this manuscript will receive your approval and be accepted for publication.

2. Comment: The submission requires further revisions with reference to both the theoretical and quantitative framework.

2. Reply: Thank you for reviewing our paper and providing valuable suggestions. I have substantially revised the original manuscript in terms of theory as well as quantity, in accordance with the comments of the reviewers.

3. Comment: Please ensure that your manuscript meets PLOS ONE's style requirements, including those for file naming.

3. Reply: Thank you for providing the style template, we have strictly followed the template provisions of the original manuscript to modify the hope that it can meet your requirements.

4. Comment: Please provide a complete Data Availability Statement in the submission form, ensuring you include all necessary access information or a reason for why you are unable to make your data freely accessible. If your research concerns only data provided within your submission, please write "All data are in the manuscript and/or supporting information files" as your Data Availability Statement.

4. Reply: Thank you for reviewing our paper and providing valuable suggestions. We highly appreciate your feedback and have made the necessary revisions accordingly. After your suggestion, we have made a data availability statement at the time of submitting the data, which includes the raw data, the source of the data and the citation. Thus, the scientific validity of this study is explained.

**Respond to the Reviewer#1's comments**

**1. Comment:** This study empirically analyses the impact of commercial credit on the urban environment by means of a spatial econometric model for China in the period 2010-2021. In doing so, spatial spillover effects and heterogeneity across regions are taken into account. The analysis finds an inverse U-shaped relationship between commercial credit and the urban environment, confirming spatial spillover effects and heterogeneity between regions. Robustness to these results is also tested. With sustainable finance gaining attention as a climate change measure, the results of this study are expected to provide useful policy recommendations. However, the explanations of previous studies, the interpretation of the results, and the multiple typos significantly undermine the academic value of this study. Improvements to these are therefore suggested as follows.

**1. Reply:** Thank you for your thorough review and valuable suggestions for my manuscript. Your meticulous examination and professional insights have greatly contributed to the improvement and enhancement of this paper in multiple aspects. Your recommendations have not only helped us delve deeper into the theoretical and methodological aspects but also made the structure and presentation of the article more rigorous and clearer.

I deeply appreciate the time and effort you have dedicated to this review, and I am pleased to have benefited from your feedback. Your suggestions have been crucial in elevating the quality and academic contribution of the paper. We hope that these improvements will enable the manuscript to better meet the journal's standards and provide valuable insights for research in the related field.

Once again, thank you for your careful review and invaluable feedback. I hope this manuscript will receive your approval and be accepted for publication.

**2. Comment:** The discovery of an inverted U-shaped relationship between business credit and the urban environment is a key result of this study. Nevertheless, in the introduction, the authors refer to previous studies on business credit improving the urban environment, but not to previous studies on its worsening the urban environment. Not only in the Literature Review in Section 2 but also in the Introduction in Section 1, an explanation should be provided regarding business credit worsening the environment and increasing pollution by citing relevant studies.

**2. Reply:** Thank you for reviewing our paper and providing valuable suggestions. We highly appreciate your feedback and have made the necessary revisions accordingly. At your suggestion, we have added the literature research on credit to the deterioration of the environment, but in view of the main purpose of the introduction is to draw out the relationship between the urban commercial credit environment and environmental pollution, for the urban commercial credit environment for the positive and negative relationship between the environmental pollution of the relevant literature research and theoretical elaboration is concentrated in the second section of this paper, the first section of the introduction mainly plays a role in drawing out the theme.

We have specifically added the following content:

Zhang and Chen found that social trust promotes green innovation and environmental protection by increasing the external environmental pressures faced by firms and enhancing their reputational motivation and environmental awareness. Narain found that good credit management encourages firms to comply with contracts and commitments, improves the quality of environmental information disclosure to build corporate image and reputation and minimize receivable risks, reduces financial constraints, stimulates innovation, and promotes green innovation to improve the environment .it can be seen that social credit plays an important role in the improvement of the urban environment. However, too strong or too weak a degree of credit regulation is detrimental to the quality of green development and must be kept under an appropriate level. (On page 3, lines 50-61).

**3. Comment:** In Table 6, the indirect effects of In(credit) and In2(credit) are just under three times more effective than their direct effects. In general, the direct effects are considered to have larger values than the indirect effects. A rational explanation should be provided for this result.

**3. Reply:** For the spatial Durbin model in the indirect effect is more than three times the direct effect of the problem, I think it is a normal empirical phenomenon, the reasons are as follows: (1) the direct effect reflects the degree of influence of the explanatory variables on the local, the indirect effect reflects the influence of the explanatory variables on the surrounding areas, is a kind of spatial spillover effect of the realization, the coefficient of the indirect effect is much larger than the coefficient of the direct effect of the problem, which means the coefficient is larger than the coefficient of the direct effect of the problem. The influence of a region's urban business credit environment on environmental pollution in its neighboring regions is much larger than its influence on environmental pollution in the region, which means that the spatial spillover effect is more obvious. (2) It is normal that the coefficient of indirect effect is larger, generally it may be manifested because of the problem of magnitude, because the variables are weighted by spatial weights, often the magnitude will be smaller, and when the explanatory variables themselves are smaller, they tend to present larger coefficient results. This can also lead to much larger coefficients for indirect effects than for direct effects. (On page 26, line 495 table 5).

**4. Comment:** The total effect of Model 1 in Table 7 is nearly 190, and the size of the value is outstanding compared to the other results. Why are the value so large?

**4. Reply:** Thank you for reviewing our paper and providing valuable suggestions. We highly appreciate your feedback and have made the necessary revisions accordingly. The results of spatial Durbin modeling using geographic distance weight matrix, economic-geographic nested matrix, and economic distance weight matrix as spatial weights, respectively, are presented in Table 6. Among them, model (1) is the result under the use of a geographic distance matrix, and its total effect appears larger compared to the other two models, which I think is normal and fully consistent with the empirical requirements. Selecting the spatial weight matrix is the first step of spatial measurement, and different choices of spatial weight matrix will bring different weighting effects on the explanatory variables, which will lead to different total effects, and the reason for the large effect of this model (1) may be that the weight matrix constructed by utilizing the factor of the distance between the cities has a greater influence on the model, but it still has not changed the characteristics of the relationship between the independent variables and the dependent variable. So it is said that the difference in the choice of weight matrix will bring different effects. However, it also shows that even if the spatial weight matrix changes, the effect of the model remains unchanged, and the use of the model to explore the relationship between the two remains robust. (On page 28, line 510 table 6). Once again, thank you for your valuable feedback. We look forward to your further comments.

**5. Comment:** The spatial weight matrix in 3.2.3 should also be accompanied by a concise explanation, rather than just stating the formula. In 3.2.3, the formula contains unnecessary small arrows, which should be deleted.

**5. Reply:** We have briefly explained the spatial weight matrix of 3.2.3 in the revised version. We have specifically added the following content:

If there is a common boundary between city and city, then note=1, otherwise=0. (On page 13, lines 272-274).

**6. Comment:** In the conclusion, the limitations of this study should be stated.

**6. Reply:** Thank you for reviewing our paper and providing valuable suggestions. We highly appreciate your feedback and have made the necessary revisions accordingly. After combing through the article, we add two limitations of this study after the policy recommendations in Section 5. Once again, thank you for your valuable feedback. We look forward to your further comments.

We have specifically added the following content:

It should be noted that this study also has some limitations. First, the limitation of data availability. The official release of the latest data of the City Business Credit Environment Index (CEI Index) is only from 2010 to 2021, so this paper only uses the panel data of 276 cities in China from 2010 to 2021 as the empirical research object. Future research could consider extending the data to prefecture-level cities after 2021, which could help validate the findings and explain any changes in trends over time, draw more specific conclusions, and make more specific policy recommendations. Second, the limitations of the study population. This study focuses only on Chinese cities, which may limit the generalizability of the findings to other contexts. Due to different regulatory, economic, and social conditions, different countries or regions may exhibit different relationships between the business credit environment and environmental pollution. Therefore, future research should expand the study sample to improve the applicability of the findings. (On page 42, line 748-On page 43, line 760).

**7. Comment:** The paper is very difficult to read due to the typos and formatting flaws as pointed out below. The entire paper should be reviewed to reduce these errors. In the references, the name of the journal is not mentioned. In Tables 3 and 4, Chinese words are included and should be corrected into English. On page 2, line 27, ‘western regions. however,’ is a period typo. In addition, there is no period typo at the end of this sentence. On page 8, line 173, ‘Exacerbating’ begins with a capital letter, even though it is in mid-sentence. On page 26, line 465, the word ‘pollution.’ does not require a period. On page 32, line 556, ‘Increasing’ starts with a capital letter, despite being in the sentence. On page 34, line 582, ‘The urban’ is capitalized at the beginning of the sentence, even though it is in the middle of the sentence.

**7. Reply:** We apologize for this error in reference formatting, and we have reorganized the literature to add the names of the journals according to the template given for the paper. thank you for pointing out the spelling errors in our manuscript. We have carefully gone through the entire document and corrected all the identified spelling mistakes. We have amended the Chinese words in Tables 2 and 3 to English and ensured that all table words have been rendered in English. we apologize for any inconvenience caused and are committed to ensuring the highest level of quality in our future submissions.

We have specifically made the following changes:

(1) Modify reference formatting and add reference sources. (On page 43, line 761-On page 45, line 873).

(2) Delete “however” and lowercase the first letter of “exacerbating”. (On page 2, line 26, On page 7, line 143)

(3) Correct the Chinese words in Tables 2 and 3. (On page 21, line 407, On page 23, line 444).

**Table 2. Global Moran Index of Environmental Pollution and** **Urban Business Credit Environment**

| Year | Environmental Pollution Index | | Urban Business Credit Environment Index | |
| --- | --- | --- | --- | --- |
|  | Moran's I | P value | Moran's I | P value |
| 2010 | 0.006 | 0.178 | 0.014 | 0.014 |
| 2011 | 0.013 | 0.013 | 0.000 | 0.575 |
| 2012 | 0.012 | 0.006 | 0.005 | 0.236 |
| 2013 | 0.023 | 0.000 | 0.002 | 0.453 |
| 2014 | 0.038 | 0.000 | -0.002 | 0.845 |
| 2015 | 0.028 | 0.000 | -0.006 | 0.711 |
| 2016 | 0.019 | 0.001 | 0.012 | 0.024 |
| 2017 | 0.012 | 0.029 | 0.029 | 0.000 |
| 2018 | -0.003 | 0.903 | 0.059 | 0.000 |
| 2019 | -0.002 | 0.824 | 0.079 | 0.000 |
| 2020 | -0.001 | 0.672 | 0.079 | 0.000 |
| 2021 | 0.002 | 0.435 | 0.079 | 0.000 |

**Table 3. Results of the LM test and LR test**

| Tests | Categorization | | Statistic | P value |
| --- | --- | --- | --- | --- |
| LM Tests | Spatial error  model（SEM） | LM-Error | 1213.794 | 0.000 |
|  |  | Robust-LM-Error | 131.899 | 0.000 |
|  | Spatial autoregressive model（SAR） | LM-Lag | 1108.816 | 0.000 |
|  |  | Robust-LM-Lag | 26.920 | 0.000 |
| LR Tests | Spatial autoregressive model（SAR）VS Spatial Durbin model (SDM) | | 19.00 | 0.0082 |
|  | Spatial error model（SEM）VS Spatial Durbin model (SDM) | | 18.20 | 0.0111 |

**Respond to the Reviewer#2's comments**

**1. Comment:** I had the opportunity to read and revised your article. The manuscript investigates the relationship between urban business credit environment and environmental pollution in 276 Chinese cities from 2010 to 2021. The study utilized the entropy method to calculate the environmental pollution index and employs the CEI index to describe the urban business credit environment. Moreover, it employs a spatial Durbin model to analyze the mechanism and transmission path of the urban business credit environment on environmental pollution.

I believe your work coherent as the Journal “PLOS ONE”, regarding an interesting topic not fully investigated in the literature until now. However, I have some suggestions and minor concerns that may be improve the quality of your work.

**1. Reply:** Thank you for your thorough review and valuable suggestions for my manuscript. Your meticulous examination and professional insights have greatly contributed to the improvement and enhancement of this paper in multiple aspects. Your recommendations have not only helped us delve deeper into the theoretical and methodological aspects but also made the structure and presentation of the article more rigorous and clearer.

I deeply appreciate the time and effort you have dedicated to this review, and I am pleased to have benefited from your feedback. Your suggestions have been crucial in elevating the quality and academic contribution of the paper. We hope that these improvements will enable the manuscript to better meet the journal's standards and provide valuable insights for research in the related field.

Once again, thank you for your careful review and invaluable feedback. I hope this manuscript will receive your approval and be accepted for publication.

**2. Comment:** Starting from a general suggestion, I recommend you to carefully read your work, since there are several typos, punctuation mistakes with too long period sentences. I list some examples that I found in the text. Lines 52-59. Line 79. Line 91-96. Line 98. Line 144. Line 214. Etc.

**2. Reply:** Thank you for pointing out the spelling errors in our manuscript. We have carefully gone through the entire document and corrected all the identified spelling mistakes. We have specifically made the following changes:

(1) Revise lines 52-59 of the initial draft as follows: Narain found that good credit management encourages firms to comply with contracts and commitments, improves the quality of environmental information disclosure to build corporate image and reputation, and minimizes receivable risks, reduces financial constraints, stimulates innovation, and promotes green innovation to improve the environment. It can be seen that social credit plays an important role in the improvement of the urban environment. (lines 53-58).

(2) Revise line 79 of the initial draft to ‘Green credit policy is the best initiative of the government to respond to green development and the construction of a social credit system’(lines 72-73). Revise line 98 of the initial draft to ‘The research on the impact of the business credit environment on environmental pollution is not systematic’(lines 90-91). Revise line 144 of the initial draft to ‘The consequence of the urban business credit environment on local environmental pollution is studied at the national level and in three regions: East, Central, and West.’(lines 123-124). Revise line 214 of the initial draft to ‘a more transparent system implementation, and a better business credit environment.’(lines 183-184).

(3)Revise lines 91-96 of the initial draft as follows: Accessing to and provision of commercial credit not only accelerates the recapitalization of companies, eases financing constraints and reduces company costs, but also brings about advances in corporate technological innovation, improves their production and pollution control technologies, and mitigates the extent of environmental pollution to a certain extent. (lines 86-90).

**3. Comment:** I suggest adjusting the Introduction section since it is too long and somewhere a bit confused. I recommend to better highlight your contribution, with the research questions, the aim and the structure of the manuscript in a logic order. Moreover, you should include some missing references: lines 52-53; lines 100-104.

**3. Reply:** We have reorganized the results of the introduction section to strictly follow the organizational structure of the introduction, literature review, research gaps, scope of the study, the contribution of the study and the paper. In addition, relevant references missing from lines 52-53 and lines 100-104 have been added.

We have specifically made the following changes: Restructuring of the original introduction. ( lines 38-133).

We have specifically added the following content；

(1)Add references missing from lines 52-53 of the original manuscript: Zhang and Chen found that social trust promotes green innovation and environmental protection by increasing the external environmental pressures faced by firms and enhancing their reputational motivation and environmental awareness. Narain found that good credit management encourages firms to comply with contracts and commitments, improves the quality of environmental information disclosure to build corporate image and reputation and minimize receivable risks, reduces financial constraints, stimulates innovation, and promotes green innovation to improve the environment. It can be seen that social credit plays an important role in the improvement of the urban environment. ( lines 50-58).

(2) Add references missing from lines 100-104 of the original manuscript: At present, research is being conducted to investigate the effects of urban business credit on environmental pollution from both macro and micro dimensions. The macro level is primarily concerned with the environmental influence of the external environment, which includes the institutional, business, and legal environments. For example, Chen, Ni, Zhu et al. (2022) found that implementing the national credit model policies helps to raise the public's understanding of the rules of the law, standardize market order, as well as enhance the effectiveness of the urban green economy. Green credit policy is the best initiative of the government to respond to green development and the construction of a social credit system, Improving green credit levels helps to foster the high-quality growth of the green economy, and raises the standard of high-quality green economy development in one area will raise the quality of high-quality green economy development in adjacent areas. Di, Li, Shen, et al. (2023) discovered that a credit system based on environmental performance helps to lower emission intensity. A favorable urban business credit climate surely results in an ideal business environment that fosters company innovation, which is more advantageous to the growth of environmentally friendly firms and the productivity of the urban green economy. Yuan, Zhang, Hu et al. (2022) found that The enhancement of the business credit environment fosters the development of the region's financial credit scale, and the resulting financial agglomeration is favorable to the environment up to a certain point. The micro level is primarily developed in terms of credit resource limits and allocation efficiency, as well as firms' green innovation potential. Accessing to and provision of commercial credit not only accelerates the recapitalization of companies, eases financing constraints and reduces company costs, but also brings about advances in corporate technological innovation, improves their production and pollution control technologies, and mitigates the extent of environmental pollution to a certain extent. The research on the impact of the business credit environment on environmental pollution is not systematic, the preceding two approaches explored the interaction between the two from the peripheral, to some extent from the macro and micro perspectives, respectively. ( lines 65-93).

**4. Comment:** The study relies on data from 276 cities over a specific period (2010-2021). If it is possible (considering availability of data) extending the analysis period beyond 2021 and incorporating more recent data could help validate the findings and account for any changes in trends over time.

**4. Reply:** Thank you for reviewing our paper and providing valuable suggestions. We highly appreciate your feedback and have made the necessary revisions accordingly. The latest official release of data for the core explanatory variable of this paper, the City Commercial Credit Environment Index (CEI Index), is only available from 2010 to 2021, and the CEI Index for cities after 2021 is not available, due to data availability limitations which is what led to the limitations of this thesis, which we hope you will understand. Once again, thank you for your valuable feedback. We look forward to your further comments.

We have specifically added the following content:

It should be noted that this study also has some limitations. First, the limitation of data availability. The official release of the latest data of the City Business Credit Environment Index (CEI Index) is only from 2010 to 2021, so this paper only uses the panel data of 276 cities in China from 2010 to 2021 as the empirical research object. Future research could consider extending the data to prefecture-level cities after 2021, which could help validate the findings and explain any changes in trends over time, draw more specific conclusions, and make more specific policy recommendations. (lines 748-754).

**5. Comment:** Although the spatial Durbin model is adequate for your research because it is useful for capturing spatial relationships, it may not fully account for all relevant variables influencing environmental pollution, such as local policy changes, industrial shifts, or unobserved regional characteristics. The study could improve by addressing potential endogeneity issues. You might consider including instrumental variables or difference-in-differences approach that could help establish causality more convincingly.

**5. Reply:** At your suggestion, we reviewed the related literature and were inspired to introduce two instrumental variables for the regression results of this paper to address the potential endogeneity problem by the instrumental variable method, which will be added to the robustness test template to enrich the credibility of the article.

We have specifically added the following content:

This study addresses the endogeneity problem that may result from two-way causality and omitted variables through two-way fixed-effects modeling of time and individuals. However, it may not be able to fully explain all the relevant variables affecting environmental pollution, such as local policy changes, industrial shifts, or unobserved regional characteristics, and this study can utilize the instrumental variable approach can alleviate the endogeneity problem. For instrumental variable selection, we first refer to Shen, Chen, and Ying (2022), which takes as an instrumental variable whether the 276 cities studied in this dissertation are the first batch of China's reform and opening-up pioneer cities and special economic zones in the 1980s (IV1), which is taken to be 1 if the city is one of China's reform and opening-up pioneer cities and special economic zones in the 1980s, and is 0 otherwise. The reason for this is that the instrumental variable needs to satisfy correlation and homogeneity, i.e., the instrumental variable should be correlated with both the endogenous explanatory variable (urban business credit environment) and uncorrelated with the disturbance term of the explanatory variable (environmental pollution). At the beginning of China's reform and opening up in the 1980s, the arrival of reform and opening up brought about an increase in social mobility in the era when resources were scarce. Against the background of the difficulty of timely follow-up of formal financial support, the scale of business credit expanded dramatically. The special economic zones (SEZs) and the coastal open cities that practiced some of the SEZ policies served as important aspects of China's open-door policy. Therefore, whether the studied city is a special economic zone or one of the first coastal open cities has an important impact on the scale of business credit, which meets the requirement of instrumental variable correlation. Moreover, the reform and opening-up are more than 40 years old, so it does not have a direct impact on the recent environmental pollution in Chinese cities, which meets the requirement of homogeneity. Second, referring to Elbahnasawy (2014), the core explanatory variable lagged one period (L. In(credit)) is chosen as the instrumental variable (IV2). This is because core explanatory variables lagged one period can not only be independent of the random disturbance term in the current period but also reflect the accumulation process of core explanatory variables in the current period (Aiello and Cardamone, 2008). We use the instrumental variables method and two-stage least squares (IV-2SLS) for estimation. The results of IV-2SLS are shown in Table 7. The coefficients of IV1 and IV2 are statistically significant, which indicates that there is a strong correlation between these instrumental and endogenous independent variables. In addition, the F-statistic test value is greater than 10 which indicates the lack of weak instrumental variables among all the selected instrumental variables. Moreover, it has been observed that the p-values of Anderson canon. Corr. LM statistics are all below 0.1, thus negating the initial hypothesis of non-identifiability. Therefore, instrumental variables IV1 and IV2 are valid. (lines 512-552).

**Table 7. Endogeneity check**

|  | (1) | (2) | (3) |
| --- | --- | --- | --- |
|  | First Stage | First Stage | Second Stage |
| VARIABLES | In(credit) | In^2^ (credit) | epi |
| IV1 | 0.0104*** | 0.0897*** |  |
|  | (5.08) | (5.14) |  |
| IV2 | 0.7584*** | 6.4739*** |  |
|  | (70.19) | (70.22) |  |
| In(credit) |  |  | 154.6552*** |
|  |  |  | (3,355.13) |
| In^2^ (credit) |  |  | -18.1073*** |
|  |  |  |  |
| lnpgdp | 0.0011 | 0.0102 | 0.0593*** |
|  | (1.10) | (1.21) | (15.87) |
| fdi | 0.1415*** | 1.2113*** | -0.0482 |
|  | (4.87) | (4.89) | (-0.43) |
| gov | -0.0011 | -0.0092 | 0.0045 |
|  | (-1.32) | (-1.27) | (1.40) |
| er | -0.4680 | -3.7925 | 9.6083*** |
|  | (-1.46) | (-1.39) | (7.88) |
| ind | 0.0003*** | 0.0020*** | -0.0025*** |
|  | (5.61) | (5.27) | (-14.71) |
| lnpd | 0.0050*** | 0.0426*** | 0.0137*** |
|  | (8.68) | (8.74) | (6.19) |
| Anderson canon. corr. LM statistic | 11.181*** | | |
| Cragg-Donald Wald F statistic | 10.595 | | |
| Observations | 3,036 | 3,036 | 3,036 |

**6. Comment:** I suggest to better describe the collection of data and the database used. For example, regarding the technological innovation, which kind of database did you used and which kind of patent?

**6. Reply:** Thank you for reviewing our paper and providing valuable suggestions. We highly appreciate your feedback and have made the necessary revisions accordingly. As per your suggestion, we have added data sources and data years and cited relevant databases to prove it. Once again, thank you for your valuable feedback. We look forward to your further comments.

We have specifically added the following content:

In this paper, the data of 276 cities in China from 2010 to 2021 are selected, and the data of the explanatory variable business credit environment are from the Blue Book of Business Credit Environment Index of Chinese Cities, which is researched and regularly published by the Business Credit Environment Index Group of Chinese Cities, and the other data are from the Statistical Yearbook of China(<https://data.cnki.net/yearBook/single?id=N2022110021>), the Environmental Statistics Yearbook of China(<https://data.cnki.net/yearBook/single?id=N2022030234>.), the Statistical Yearbook of Science and Technology of China(<https://data.cnki.net/yearBook/single?nav=%E6%95%99%E8%82%B2%E7%A7%91%E6%8A%80&id=N2023030111> ),the Financial Yearbook of China(<https://data.cnki.net/yearBook/single?id=N2022040012>), the EPS data platform, and statistical yearbooks of China's prefecture-level cities. The data that use the latitude and longitude coordinates of each prefecture-level city in China to represent the distance between each prefecture-level city come from the 1:400,000 topographic database of the National Fundamental Geographic Information System (NFGIS). (lines 368-383).

In addition, this paper describes some of the data availability notes. ( lines 890-938).

**7. Comment:** Moreover, the study focuses only on Chinese cities, which may limit the generalizability of the findings to other contexts. Different countries or regions might exhibit different relationships between business credit environments and environmental pollution due to varying regulatory, economic, and social conditions. Therefore, I suggest considering this aspect mentioning it at the end of the manuscript in the Conclusion section as future directions and further investigations.

**7. Reply:** This is a limitation of the study population in this paper. This study focuses only on Chinese cities, which may limit the generalizability of the findings to other contexts. Due to different regulatory, economic, and social conditions, different countries or regions may exhibit different relationships between the business credit environment and environmental pollution. After your point of view, we put the inclusion of limitations after policy recommendations as a future direction and further investigation.

We have specifically added the following content:

It should be noted that this study also has some limitations. ……Second, the limitations of the study population. This study focuses only on Chinese cities, which may limit the generalizability of the findings to other contexts. Due to different regulatory, economic, and social conditions, different countries or regions may exhibit different relationships between the business credit environment and environmental pollution. Therefore, future research should expand the study sample to improve the applicability of the findings. (lines 755-760).

**8. Comment:** The article suggests that improving the urban business credit environment can eventually lead to reduced environmental pollution. However, the policy recommendations could be more detailed, outlining specific measures that cities can take to balance business credit improvements with environmental sustainability.

**8. Reply:** We have now simplified and distilled the conclusions and policies in Section 5 and have elaborated the policy recommendations in more detail.

We have specifically added the following content:

Based on the above conclusions, this paper puts forward the following recommendations:

(1) The government should establish a system of environmental credit rules that matches green development. For example, the government should gradually incorporate environmental indicators, such as environmental protection inputs, emission control, and resource use efficiency, into the business credit evaluation system to ensure that the credit evaluation of business entities matches their environmental performance. It can guide enterprises toward a more environmentally friendly and sustainable direction, contributing to the realization of a green economy and sustainable development goals.

(2) Implement a dynamic and coordinated linkage development strategy for building a good urban credit system between regions. Strategies such as regional synergy, regional cooperation, and regional integration between regions should further strengthen credit information fusion and sharing, interconnection and mutual checking, organize cross-regional credit construction interaction, promote credit supervision integration, establish and improve cross-regional coordination and co-promotion mechanisms, guide the flow and proliferation of relevant resources to the central and western regions, and achieve the purpose of narrowing the differences in the level of urban business credit between regions, so as to unleash the business credit environment and reduce environmental pollution. dividend of reducing environmental pollution.

(3) The government should improve the financial ecological environment and optimize the business credit environment to effectively increase regional financial credit support to promote the improvement of the regional financial development level. Secondly, the local government should use the combination of financial subsidy policy, tax policy, and industrial policy to attract innovative enterprises, further stimulate innovation live, improve the level of urban green technology innovation, and achieve the effect of emission reduction. (lines 720-747).

**9. Comment:** Finally, I suggest mentioning the limitation/limits of the study.

**9. Reply:** Thank you for reviewing our paper and providing valuable suggestions. We highly appreciate your feedback and have made the necessary revisions accordingly.

After combing through the article, we add two limitations of this study after the policy recommendations in Section 5. Once again, thank you for your valuable feedback. We look forward to your further comments.

We have specifically added the following content；

It should be noted that this study also has some limitations. First, the limitation of data availability. The official release of the latest data of the City Business Credit Environment Index (CEI Index) is only from 2010 to 2021, so this paper only uses the panel data of 276 cities in China from 2010 to 2021 as the empirical research object. Future research could consider extending the data to prefecture-level cities after 2021, which could help validate the findings and explain any changes in trends over time, draw more specific conclusions, and make more specific policy recommendations. Second, the limitations of the study population. This study focuses only on Chinese cities, which may limit the generalizability of the findings to other contexts. Due to different regulatory, economic, and social conditions, different countries or regions may exhibit different relationships between the business credit environment and environmental pollution. Therefore, future research should expand the study sample to improve the applicability of the findings. (lines 748-760).

**Respond to the Reviewer#3's comments**

**1. Comment:** Review of the manuscript "A study of the impact of urban business credit environment on environmental pollution" (PONE-D-24-15225). The study highlights a positive spatial association between urban pollution and commercial credit indices in China; with an inverted "U" shape relationship indicating that improving the urban business credit environment can reduce pollution once a threshold is reached. Regional variations exist in the impact of business credit on environmental deterioration, emphasizing the need for tailored strategies to strengthen commercial credit systems and promote environmental sustainability in different regions.

**1. Reply:** Thank you for your thorough review and valuable suggestions for my manuscript. Your meticulous examination and professional insights have greatly contributed to the improvement and enhancement of this paper in multiple aspects. Your recommendations have not only helped us delve deeper into the theoretical and methodological aspects but also made the structure and presentation of the article more rigorous and clearer.

I deeply appreciate the time and effort you have dedicated to this review, and I am pleased to have benefited from your feedback. Your suggestions have been crucial in elevating the quality and academic contribution of the paper. We hope that these improvements will enable the manuscript to better meet the journal's standards and provide valuable insights for research in the related field.

Once again, thank you for your careful review and invaluable feedback. I hope this manuscript will receive your approval and be accepted for publication.

**2. Comment:** Line 53 – 55: Previous research has demonstrated the significant influence social credit plays in improving the urban environment. Please cite the related research articles for in-depth understanding.

**2. Reply:** Thank you for reviewing our paper and providing valuable suggestions. We highly appreciate your feedback and have made the necessary revisions accordingly. We have added relevant research literature to flesh out our points in the appropriate places. Once again, thank you for your valuable feedback. We look forward to your further comments.

We have specifically added the following content:

Zhang and Chen found that social trust promotes green innovation and environmental protection by increasing the external environmental pressures faced by firms and enhancing their reputational motivation and environmental awareness. Narain found that good credit management encourages firms to comply with contracts and commitments, improves the quality of environmental information disclosure to build corporate image and reputation and minimize receivable risks, reduces financial constraints, stimulates innovation, and promotes green innovation to improve the environment. It can be seen that social credit plays an important role in the improvement of the urban environment. (lines 50-58).

**3. Comment:** Section 1 Introduction: The section is quite lengthy and splitting the section into various sub-sections such as Introduction, Literature Review, Research gap, Scope of the study, and Research questions will be more helpful. In the introduction, you need to connect the current state of research to your paper goals. Please follow the literature review by a clear and concise state of the art analysis. This should clearly show the knowledge gaps identified and link them to your paper goals. Also, please provide the organization of the paper in the last paragraph of the introduction.

3. Reply: We have reorganized the results of the introduction section to strictly follow the organizational structure of the introduction, literature review, research gaps, scope of the study, the contribution of the study and the paper. (lines 38-133). We also provide the organization of the paper in the last paragraph of the introduction. (lines 129-132).

We have specifically made the following changes: Restructuring of the original introduction.

Strengthening pollution prevention and ecological civilization construction is the primary priority for achieving the optimization of urban economic structure and green sustainable development. However, in the context of China's present harsh development model, which prioritizes speed above quality, this has resulted in poor environmental quality, ecological destruction, and a major public health danger situation. At the moment, many scholars are primarily concerned with focusing on government behavior, institutional factors, the legal environment, and other formal institutions that can play a restraining role on urban environmental conditions, but as China has entered the "credit economy era," the main transaction form of the market has also changed to one dominated by credit transactions. To improve the morality and integrity of the entire society, a social credit system was started to be built in 2003. Scholars have placed a great emphasis on trust as the foundation of the informal system, Zhang and Chen found that social trust promotes green innovation and environmental protection by increasing the external environmental pressures faced by firms and enhancing their reputational motivation and environmental awareness. Narain found that good credit management encourages firms to comply with contracts and commitments, improves the quality of environmental information disclosure to build corporate image and reputation and minimize receivable risks, reduces financial constraints, stimulates innovation, and promotes green innovation to improve the environment.It can be seen that social credit plays an important role in the improvement of the urban environment.

However, too strong or too weak a degree of credit regulation is detrimental to the quality of green development and must be kept under an appropriate level.as the foundation of the modern credit system and the urban credit system, is also worth paying attention to whether business credit can play an environmental governance effect and promote the sustainable development of China's economy.

At present, research is being conducted to investigate the effects of urban business credit on environmental pollution from both macro and micro dimensions. The macro level is primarily concerned with the environmental influence of the external environment, which includes the institutional, business, and legal environments. For example, Chen, Ni, Zhu et al. (2022) found that implementing the national credit model policies helps to raise the public's understanding of the rules of the law, standardize market order, as well as enhance the effectiveness of the urban green economy. Green credit policy is the best initiative of the government to respond to green development and the construction of a social credit system, Improving green credit levels helps to foster the high-quality growth of the green economy, and raises the standard of high-quality green economy development in one area will raise the quality of high-quality green economy development in adjacent areas. Di, Li, Shen, et al. (2023) discovered that a credit system based on environmental performance helps to lower emission intensity. A favorable urban business credit climate surely results in an ideal business environment that fosters company innovation, which is more advantageous to the growth of environmentally friendly firms and the productivity of the urban green economy. Yuan, Zhang, Hu et al. (2022) found that The enhancement of the business credit environment fosters the development of the region's financial credit scale, and the resulting financial agglomeration is favorable to the environment up to a certain point. The micro level is primarily developed in terms of credit resource limits and allocation efficiency, as well as firms' green innovation potential. Accessing to and provision of commercial credit not only accelerates the recapitalization of companies, eases financing constraints and reduces company costs, but also brings about advances in corporate technological innovation, improves their production and pollution control technologies, and mitigates the extent of environmental pollution to a certain extent. The research on the impact of the business credit environment on environmental pollution is not systematic, the preceding two approaches explored the interaction between the two from the peripheral, to some extent from the macro and micro perspectives, respectively.

Although the above literature from the macro and micro perspectives on the relationship between business credit environment and environmental pollution, few studies directly analyze the relationship between urban business credit and environmental pollution, and the first law of geo-economics points out that everything is related to other things, the closer the distance between the two things, the stronger the relevance of this relationship. Although China has a vast territory but uneven distribution of resources, which will inevitably lead to differences in the environmental situation of each region, the spatial spillover effect needs to be considered when exploring the urban business credit environment on environmental pollution. Therefore, this study takes 276 cities in China as an example and deeply analyzes the impact of the urban business credit environment on environmental pollution from both theoretical and empirical perspectives. At the methodological level, spatial econometrics is utilized to explore the spatial spillover effect of the urban business credit environment on environmental pollution. The direct, indirect, and total effects are then further analyzed to provide the necessary theoretical support for the rational formulation and effective implementation of environmental protection policies in China.

This study adds three minor contributions to the literature in this field. (1) By creating a model that places environmental pollution and the urban business credit environment within the same research framework, this paper analyzes the mechanism by which the urban business credit environment influences environmental pollution directly. It also analyzes the direction of the role of the urban business credit environment and the magnitude of its influence on environmental pollution, to uncover the significance of the urban business credit environment. (2) To address the shortcomings, the article builds a spatial measurement model by incorporating the spatial lag of environmental pollution into the explanatory variables of the model. It also examines the effects of the urban business credit environment on adjacent areas from a spatial spillover perspective, expanding our knowledge of spatial correlation and offering resources for further research on inter-regional environmental pollution management. (3) The consequence of the urban business credit environment on local environmental pollution is studied at the national level and in three regions: East, Central, and West. The influence of regional variability in the urban business credit environment on environmental pollution is investigated, and the magnitude of this effect is verified in various regions. (4) Investigate the transmission pathways of urban business credit on environmental pollution through the lens of urban financial development and technology innovation.

The rest of the paper is organized as follows:(1) Section 2 is devoted to the theory and hypotheses; (2) Section 3 details the research design, including model development, description of variables, and data sources and descriptions; (3) Section 4 contains the results of the empirical analyses and a discussion; and (4) Section 5 provides conclusions and recommendations. ( lines 38-133).

**4. Comment:** Section 4 Empirical results and analysis: Would you consider splitting the fourth section, into two parts, Results and Discussion? At present, the length of the results section is too long, which is not conducive to grasping the key points.

**4. Reply:** We make new adjustments to the empirical results and analysis sections in Section 4 to avoid overly long paragraphs and confusing structure. The original section is divided into two parts: results and discussion; the original results section combines the original benchmark regression and spatial econometric regression results; the original theory part of the mediated effects model is put into the theory and assumptions part of section 2; the current results section only deals with the analysis of empirical results, and the discussion section only deals with the analysis and discussion of the results, with no hybridization between the two. (lines 393-699).

**5. Comment:** Section 5 Conclusion and policy implications are too long, and it is recommended that the main conclusions be distilled and streamlined.

**5. Reply:** Thank you for reviewing our paper and providing valuable suggestions. We highly appreciate your feedback and have made the necessary revisions accordingly. We have now simplified and distilled the conclusions and policies in Section 5 and have elaborated the policy recommendations in more detail. Once again, thank you for your valuable feedback. We look forward to your further comments.

We have specifically made the following changes, modify the following:

(1)5.1 Conclusion

The environmental pollution problem has become the main weakness of the current ecological civilization construction and high-quality economic development, and the informal system of urban business credit may provide an opportunity to solve this problem. Therefore, based on the panel data of 276 prefecture-level cities in China from 2010 to 2021, this paper analyzes the influence of urban business credit environment on environmental pollution and its spatial effect from various angles, such as regional division and spatial effect decomposition, with the help of spatial Durbin model. The main conclusions are as follows (1) There is a significant positive spatial correlation between China's urban pollution index and urban business credit index, and the degree of spatial agglomeration of the two has been increasing over time. (2) There is an inverted “U” shape relationship between the urban business credit environment and environmental pollution and a significant spatial spillover effect. (3) The influence of the business credit environment on environmental pollution is regionally heterogeneous. The role of the eastern and western regions is consistent with the overall situation, but in the central region, there is a certain degree of differentiation. (4) The non-linear impact of the business credit environment on environmental pollution is mainly transmitted through two channels: the level of urban financial development and the level of urban innovation.

(2)5.2 Policy recommendations

Based on the above conclusions, this paper puts forward the following recommendations:

(1) The government should establish a system of environmental credit rules that matches green development. For example, the government should gradually incorporate environmental indicators, such as environmental protection inputs, emission control, and resource use efficiency, into the business credit evaluation system to ensure that the credit evaluation of business entities matches their environmental performance. It can guide enterprises toward a more environmentally friendly and sustainable direction, contributing to the realization of a green economy and sustainable development goals.

(2) Implement a dynamic and coordinated linkage development strategy for building a good urban credit system between regions. Strategies such as regional synergy, regional cooperation, and regional integration between regions should further strengthen credit information fusion and sharing, interconnection and mutual checking, organize cross-regional credit construction interaction, promote credit supervision integration, establish and improve cross-regional coordination and co-promotion mechanisms, guide the flow and proliferation of relevant resources to the central and western regions, and achieve the purpose of narrowing the differences in the level of urban business credit between regions, so as to unleash the business credit environment and reduce environmental pollution. dividend of reducing environmental pollution.

(3) The government should improve the financial ecological environment and optimize the business credit environment to effectively increase regional financial credit support to promote the improvement of the regional financial development level. Secondly, the local government should use the combination of financial subsidy policy, tax policy, and industrial policy to attract innovative enterprises, further stimulate innovation live, improve the level of urban green technology innovation, and achieve the effect of emission reduction. (lines 700-747).

**6. Comment:** The sub-section 5.2 Policy Recommendations: It can be included in the separate sub-section into Discussion section.

**6. Reply:** Regarding the addition of the policy recommendations to the discussion section in subsection 5.2, since the discussion section in part 4 is a discussion of the empirical results, once added it will make the whole part 4 too long, affecting the reader's perception and causing confusion in the results, so we refine and fine-tune the policy recommendations section, and finally place it after the conclusion in section 5.

We have specifically made the following changes, modify the following:

Based on the above conclusions, this paper puts forward the following recommendations:

(1) The government should establish a system of environmental credit rules that matches green development. For example, the government should gradually incorporate environmental indicators, such as environmental protection inputs, emission control, and resource use efficiency, into the business credit evaluation system to ensure that the credit evaluation of business entities matches their environmental performance. It can guide enterprises toward a more environmentally friendly and sustainable direction, contributing to the realization of a green economy and sustainable development goals.

(2) Implement a dynamic and coordinated linkage development strategy for building a good urban credit system between regions. Strategies such as regional synergy, regional cooperation, and regional integration between regions should further strengthen credit information fusion and sharing, interconnection and mutual checking, organize cross-regional credit construction interaction, promote credit supervision integration, establish and improve cross-regional coordination and co-promotion mechanisms, guide the flow and proliferation of relevant resources to the central and western regions, and achieve the purpose of narrowing the differences in the level of urban business credit between regions, so as to unleash the business credit environment and reduce environmental pollution. dividend of reducing environmental pollution.

(3) The government should improve the financial ecological environment and optimize the business credit environment to effectively increase regional financial credit support to promote the improvement of the regional financial development level. Secondly, the local government should use the combination of financial subsidy policy, tax policy, and industrial policy to attract innovative enterprises, further stimulate innovation live, improve the level of urban green technology innovation, and achieve the effect of emission reduction. (lines 720-747)

**Respond to the Reviewer#4's comments**

**1. Comment:** The Manuscript titled A Study of the Impact of Urban Business Credit Environment on Environmental Pollution (PONE-D-24-15225) tries to investigate the impact based on 276 cities in China.

**1. Reply:** Thank you for your thorough review and valuable suggestions for my manuscript. Your meticulous examination and professional insights have greatly contributed to the improvement and enhancement of this paper in multiple aspects. Your recommendations have not only helped us delve deeper into the theoretical and methodological aspects but also made the structure and presentation of the article more rigorous and clearer.

I deeply appreciate the time and effort you have dedicated to this review, and I am pleased to have benefited from your feedback. Your suggestions have been crucial in elevating the quality and academic contribution of the paper. We hope that these improvements will enable the manuscript to better meet the journal's standards and provide valuable insights for research in the related field.

Once again, thank you for your careful review and invaluable feedback. I hope this manuscript will receive your approval and be accepted for publication.

**2. Comment:** The draft is loosely framed and lacks the discussion section completely

**2. Reply:** After reading your suggestions, we re-framed the paper in a big way and added a discussion section. thank you for your valuable feedback. We look forward to your further comments. We have specifically added the following content:

Discussion: As shown in the regression results in Table 4, there is an inverted U-shaped relationship between the urban business credit environment and environmental pollution, that is, before crossing the inflection point, the increase of urban business credit environment will aggravate the environmental pollution, but after reaching the inflection point value, the rise of urban business credit environment will reduce the environmental pollution. It shows that the urban business credit environment has a positive impact on changing environmental conditions. Analyze the reason, when the size of business credit is small, along with its growth China's environmental pollution situation intensifies continuously. At this stage, the business credit environment is a “poison” for the urban environment, because business credit is a kind of public credit with a higher degree of standardization, which is more subject to informal constraints such as reputation and morality, and excessive transaction costs are not conducive to the sustained and healthy operation of enterprises and may reduce the utilization of resources and increase the emission of pollution, which is not conducive to the urban environment. When the business credit environment reaches a critical value, it will play the role of “antidote”, which is likely to improve urban pollution. At this time, along with the development of green finance, the formal financial credit support for the green economy continues to increase, but after reaching a certain loan size, under the constraints of legal deposit reserves, deposit absorption, and other factors, the difficulty of formal financial credit increases steeply; as an important backup funding channel outside the formal financial credit, business credit to alleviate the financing constraints highlights the role of business credit to enhance the regional environmental pollution. important role.

The effect decomposition results of the spatial Durbin model in Table 5 show that the direct, indirect, and total effects of the primary term of the urban business credit environment are all significantly positive, indicating that the growth of the urban business credit environment will exacerbate the urban environmental pollution before the inverted U-shaped inflection point is crossed, and there is a positive spatial spillover to the neighboring areas, which will exacerbate the surrounding environmental pollution. The positive spatial spillover to the neighboring areas will aggravate the environmental pollution in the surrounding areas. According to the secondary term of urban business credit environment, the direct effect, indirect effect, and total effect are all significant and the coefficient is negative, which indicates that there is an inverted “U” curve relationship between urban business credit environment and environmental pollution, and the effect of urban environmental pollution aggravating environmental pollution is not always increasing, but there is a certain threshold. This result may be because after the urban business credit environment reaches a certain height, the improvement of the urban business credit level is conducive to the local enterprises to alleviate the problem of financing constraints, improve the infrastructure and public services in the urban area, and prompt the enterprises to carry out technological innovation and promote green development. Under the regional transmission mechanism of the “learning effect”, the business credit atmosphere in the region can be radiated to the neighboring regions and improve the environmental conditions of the neighboring cities.

According to the mediation effect regression results in Table 8 and Table 9, in the short term, the strengthening of the business credit environment will bring about a reduction in the level of regional financial development and scientific and technological innovation capacity, which will exacerbate the environmental pollution, and with the further acceleration of the business credit environment in the city, the transformation of the financial development awareness and concepts, as well as the application of advanced technology, will lead to the development of the regional financial industry and the enhancement of the capacity of innovation, which will form a constraint on the environmental pollution. One of the reasons may be that of China's lagging financial industry system and system, the rough development in the early stage will bring about environmental degradation, with the formalization of finance, the financial industry development of the green development of the matching degree is gradually enhanced, the business credit environment will reduce environmental pollution. For green innovation, before reaching the critical point, subject to financing constraints, the innovation ability of enterprises is weak, and cannot take into account the protection of the environment, with the construction of the system and the credit joint reward and punishment mechanism is constantly sound, under the role of the reputation effect mechanism, it will accelerate the enterprise business expansion and technological innovation incentives, and the green innovation ability has a positive impact on the reduction of environmental pollution.

Table 10 Regional heterogeneity analysis results can be seen in the East, Central, and West three parts of the region in the urban business credit environment on environmental pollution there is a significant negative spatial effect. And in the east and west of the urban business credit environment on environmental pollution shows an inverted “U” non-linear relationship between the urban business credit environment, the urban business credit environment indirect effect is significant that the urban business credit environment and environmental pollution has a spatial spillover effect, this inverted “U” relationship is not significant in the central cities. This inverted “U”-shaped relationship is not significant in central cities. This may be due to the fact that, compared with the central region, the eastern region has a higher degree of capital abundance for development, and the market operation mechanism is more soundness. Before the business credit environment reaches the critical point, there is a substitution relationship between business credit and lower-cost formal finance, and the financing constraints faced by enterprises are generally weaker. However, after the business credit environment reaches the tipping point, there is a complementary relationship between business credit and formal finance. Enterprises are gradually emphasizing green production, reducing energy consumption, and enhancing environmental protection. In the western region, where the primary industry is the main industry and the level of environmental pollution is low, once business credit is used to obtain funds, it is conducive to prompting enterprises to develop or purchase green technologies to further strengthen the prevention and control of environmental pollution. This provides another way of thinking to better understand the Western development for green transformation. While the central region of the city credit level development is slow, industrial development is still in transition, the degree of marketization is weak, and the central city of industrialization is higher, but brought serious environmental pollution, so the urban business credit environment of urban environmental pollution is not obvious to reduce the effect of enhancement of the role of the lack of. It can be seen that the impact of the urban business credit environment on environmental pollution varies from region to region.

To summarize, the influence of the urban business credit environment on urban environmental pollution has an inverted “U” shape relationship both in the whole and in the region. (lines 603-699).

**3. Comment:** The data source and year of data is missing (no citations provided).

**3. Reply:** As per your suggestion, we have added data sources and data years and cited relevant databases to prove it. We have specifically added the following content:

In this paper, the data of 276 cities in China from 2010 to 2021 are selected, and the data of the explanatory variable business credit environment are from the Blue Book of Business Credit Environment Index of Chinese Cities, which is researched and regularly published by the Business Credit Environment Index Group of Chinese Cities, and the other data are from the Statistical Yearbook of China(<https://data.cnki.net/yearBook/single?id=N2022110021>), the Environmental Statistics Yearbook of China(<https://data.cnki.net/yearBook/single?id=N2022030234>.), the Statistical Yearbook of Science and Technology of China(<https://data.cnki.net/yearBook/single?nav=%E6%95%99%E8%82%B2%E7%A7%91%E6%8A%80&id=N2023030111> ),the Financial Yearbook of China(<https://data.cnki.net/yearBook/single?id=N2022040012>), the EPS data platform, and statistical yearbooks of China's prefecture-level cities. The data that use the latitude and longitude coordinates of each prefecture-level city in China to represent the distance between each prefecture-level city come from the 1:400,000 topographic database of the National Fundamental Geographic Information System (NFGIS). (lines 368-383).

In addition, this paper describes some of the data availability notes. ( lines 890-938).

**4. Comment:** Spatial mapping of the 276 selected cities can really add value to the paper. The authors should adopt appropriate methods and tools for the same.

**4. Reply:** We used ArcMap software to map the spatial and temporal distribution of 276 prefecture-level cities in 2010 and 2021 (Figures 2 and 3) to observe the changes in environmental pollution and urban business credit environment in each city and then described their spatial characteristics based on the charts. We have specifically added the following content:

In order to further analyze the spatial distribution of the urban business credit environment and environmental pollution in 276 prefecture-level cities, this paper used ArcMap 10.7 to map the spatial distribution of the urban business credit environment and environmental pollution in 2010 and 2021 (shown in Figures 2 and 3).

**Fig 2. Spatial characteristics of environmental pollution in 2010 and 2021.**

**Fig 3. Spatial characteristics of the urban business credit environment in 2010 and 2021.**

Figure 2 shows the environmental pollution in 276 cities in 2010 and 2021. This figure indicates the evolution of environmental pollution, and its severity is indicated by the color shades, with darker colors indicating more severe environmental pollution. During the period 2010-2021, the overall pollution situation in the whole of China has decreased. Overall, in terms of spatial distribution, this indicates that pollution is more severe in cities in the central and western parts of the country than in the eastern parts of the country. This may be due to the implementation of the “Western Development” strategy, the Western region vigorously develops the secondary industry, but at the same time, it also has an impact on the environment.

As shown in Figure 3, the urban business credit environment index of 276 prefecture-level cities is divided into three levels. From a spatial point of view, the number of cities with medium and high levels of urban business credit environment increased significantly from 2010 to 2021, and most of the cities in the Southeast Coastal Region have high levels of business credit, while most of the cities in the Central and Western Regions are at a lower level. This also fully demonstrates that the business credit level of prefecture-level cities in China has regional differences. (lines 409-430).

**5. Comment:** A Methodology chart is not included in the paper …which is a must.

**5. Reply:** I have improved the methodology in the revised draft and added a methodology chart (Figure 1) to show my methodological process more clearly. We have specifically added the following content:

1. Spatial Durbin model

Interregional “siphon effects” and “spillover effects” often lead to the interpenetration and radiation of environmental pollution and economic activities between cities…….

The first step in establishing a spatial Durbin model is to select a suitable spatial weight matrix, which is generally considered to be of four types: 0-1 spatial weight matrix, geographic distance spatial weight matrix, economic distance spatial weight matrix, and economic-geographic nested spatial weight matrix. The four types of weight matrices are as follows:

If there is a common boundary between city and city, then note=1, otherwise=0.

indicates the geographic distance between the city and city in terms of the latitude and longitude of the city center.

and represent the GDP per capita of City and City, respectively.

Among them.indicates the geographic distance between the city and the city in terms of the latitude and longitude of the city center.denotes the average GDP per capita of the city during the study period, denotes the average GDP per capita of the city during the study period, and、 denotes the weights given to geographic and economic distance,taking the value of 0.5 in this study.

Selecting Moran's I was used to performing the spatial autocorrelation test before estimating the spatial model's parameters. Moran's I have a value between -1 and 1, the stronger the autocorrelation, the closer the values are to -1 and 1. A positive correlation is shown by a Moran's value larger than 0, and a negative correlation is indicated by a value smaller than 0:

Where,、denotes the specific observed values of the environmental pollution index and urban business credit environment index of a region in the year, respectively, and is the total number of regional samples.

Consequently, the spatial Durbin model based on model (1) is constructed in this study using the 0-1 spatial weight matrix(), and the model's specific form is as follows：

Where denotes region;denotes time,and distributions denote the urban business credit environment index and the quadratic term of city business credit environment and denote the level of environmental pollution;denote the relevant control variables;denotes spatial weights;denotes time fixed effects;denotes individual fixed effects; and is the random error term. Where denotes the interaction between the dependent variable and the neighboring unit dependent variable,is the spatial autoregressive coefficient,is a constant term, and is a fixed unknown parameter.

2. Mediated effects model

In order to verify the two conduction paths of technological innovation level (conduction path Ⅰ) and financial development level (conduction path Ⅱ) to explore the mechanism of business credit environment affecting environmental pollution, the following model is established to carry out further analysis:

Where characterizes the possible path of business credit environment affecting environmental pollution,characterizes the degree of its influence,characterizes the level of business credit environment in the region in the first year, and represents the control variables.

Combining the above theoretical analysis and modeling process, the research content and methodology are shown in Figure 1. (lines 255-320).

**Figure 1. Methodological framework diagram**

**6. Comment:** The same theme is repeated several times in the paper and reiterated without proper citations.

**6. Reply:** Thank you for reviewing our paper and providing valuable suggestions. We have reorganized it at your suggestion to avoid repetition of themes and to add and aptly cite relevant literature. We highly appreciate your feedback and have made the necessary revisions accordingly. Once again, thank you for your valuable feedback. We look forward to your further comments.

**7. Comment:** Only 26 references make the whole research very subjective.

**7. Reply:** As suggested by the reviewer, we have checked the literature carefully and added more references to the revised manuscript. The current bibliography has been supplemented with forty-eight references, mainly used to support the theory and methodology of the thesis. (lines 761-873).

**8. Comment:** This paper needs a complete overhauling. A major revision of the draft is essential.

**8. Reply:** Thank you for reviewing our paper and providing valuable suggestions. We highly appreciate your feedback and have made the necessary revisions accordingly. Once again, thank you for your valuable feedback. We look forward to your further comments.
